# Effect of oxygen and nanoparticles on human skin and colon cells exposed to synchrotron-based X-ray FLASH beams

**DOI:** 10.1107/S160057752600398X

**Published:** 2026-05-07

**Authors:** Moshi Geso, Matthew Cameron, Tom Ffrench, Helen Forrester, Rod Lynch, Masao Nakayama, William Patterson, Wan Nordiana Rahman, Duncan Butler, Terrence J. Piva

**Affiliations:** a School of Health and Biomedical Science RMIT University Bundoora Victoria3083 Australia; b IMBL, ANSTO-Australian Synchrotron, Clayton, Victoria3168, Australia; c Radiotherapy Department University Hospital Geelong Geelong Victoria3220 Australia; d Centre for Medical Radiation Physics University of Wollongong Wollongong New South Wales2500 Australia; e Division of Radiation Oncology Kobe University Graduate School of Medicine Kobe Hyogo650-0017 Japan; f ICON Cancer Centre, Waurn Ponds, Victoria3216, Australia; g Department of Applied Physics, Faculty of Science and Technology Universiti Kebangsaan Malaysia Bangi Selangor Malaysia; h Australian Radiation and Protection and Nuclear Safety Agency, Yallambie, Victoria3085, Australia; Australian Synchrotron, Australia

**Keywords:** FLASH effect, X-ray radiation, synchrotron, cell viability, nanoparticles, hypoxia, skin cells, colon cells

## Abstract

FLASH radiotherapy involves delivering relatively high radiation doses at ultra-high dose rates (UHDRs) that are several orders of magnitude greater than those used in conventional radiotherapy (40 Gy s^−1^ versus 0.5–5 Gy min^−1^, respectively). Previous *in vivo* studies have shown that doses delivered with such UHDRs result in significant tumour killing while having less effect on normal tissues. Most of these *in vivo* studies were based on the use of charged particles such as electrons and protons. In this study we exposed cells [human epidermal melanocytes (HEM), MM96L melanoma cells, CCD841 colon epithelial cells and CaCo2 colorectal adenocarcinoma cells] grown *in vitro* to synchrotron-based X-ray beams delivered at either low dose rates or UHDRs to validate the FLASH effect. The FLASH effect that has been reported to occur under hypoxic conditions was also investigated using HEM and MM96L cells. Significant cell killing was observed at 48 h post-irradiation, when the cells were exposed to high dose (≥10 Gy) UHDRs compared with low dose rate beams in both groups of cells. MM96L melanoma cells were ∼10% less resistant to UHDR than were HEM cells. A similar result was observed in CCD841 and CaCo2 cells. When the hypoxic melanocytes were exposed to (≥10 Gy) UHDRs a minimal loss of cell viability was observed; however, when hypoxic MM96L cells were irradiated, significant cell losses were observed. These results show that a FLASH effect is evident in these skin and colon cells. Moreover, when MM96L melanoma cells were pretreated with 1 m*M* gold nanoparticles and exposed to 10 Gy UHDR X-rays there was a 50% dose enhancement observed where only 15% was observed at low dose rates.

## Abstract 2

Most cancer patients will be treated by radiotherapy, and recent findings suggest that delivering radiation at ‘FLASH’ ultra-high dose rates reduces damage to healthy tissues while still damaging the tumour. This project experimentally validates the reduced healthy tissue damage of FLASH radiotherapy on cells using synchrotron-based beams, which will support future clinical trials and ultimately leading to its application in cancer treatment.

## 1. Introduction

Cancer along with heart disease are currently the main causes of death worldwide (Bray *et al.*, 2021 ▸). In Australia in 2022, some 162000 cases were diagnosed along with 50000 deaths (Cancer Australia, 2025 ▸). The four main types of cancer diagnosed in Australia are prostate, breast, melanoma and colorectal cancers. In treating advanced cancers, multidisciplinary treatments such as surgical resection, chemotherapy, radiotherapy, immunotherapy and combination therapy have been used (Granata *et al.*, 2025 ▸; Pail *et al.*, 2025 ▸; Tiwari *et al.*, 2025 ▸). Among these, radiotherapy (RT) has long been an effective method for treating these tumours, and it is estimated that ∼50% of cancer patients require RT, either as a primary treatment or in combination with other therapies. The main objective of enhancing radiotherapy is to maximize the destruction of tumour tissue within the irradiated field while minimizing harm to the surrounding healthy tissue. Reducing and preventing radiation-related side effects because of radiotherapy is crucial in improving its efficacy as well as the patient’s well being.

New radiotherapeutic treatment approaches for solid cancers/tumour have been developed and evaluated to achieve a differential response between targeted tumour cells and the surrounding tissue. A notable advancement in radiotherapy is currently being investigated where the radiation dose is administered at an ultra-high dose rate (UHDR) of >40 Gy s^−1^ which is known as FLASH radiotherapy (FLASH-RT) (Montay-Gruel *et al.*, 2017 ▸). This method has shown similar efficacy to conventional radiotherapy (CONV-RT) (∼0.03 Gy s^−1^) in controlling tumour progression, while significantly reducing healthy tissue toxicity commonly seen in standard conventional treatments based on low dose rate beams.

The FLASH effect has been validated in preclinical studies using both electron and proton beams (Alhaddad *et al.*, 2024 ▸). However, the biological mechanisms elicited by FLASH-RT are still not well understood. In a landmark *in vivo* study, Favaudon *et al.* (2014 ▸) demonstrated that, compared with CONV-RT (0.03 Gy s^−1^), FLASH-RT (>40 Gy s^−1^) significantly reduced radiation-induced lung fibrosis in mice while maintaining its effectiveness against lung tumours. This phenomenon is now known as the ‘FLASH effect’. The first clinical trial using FLASH-RT was undertaken in 2019 on a 75 year-old patient with recurrent cutaneous T-cell lymphoma affecting both elbows. One elbow was irradiated with FLASH-RT, the other with CONV-RT (Bourhis *et al.*, 2019 ▸). Researchers assessed acute reactions and followed the patient for two years, finding similar outcomes in tumour control and side effects between the two treatment methods indicating no worse outcomes for ultra-high dose delivery.

At present, due to lack of data there are no defined cohorts that would benefit from FLASH-RT. First, the evaluation of patient eligibility for FLASH-RT remains inadequate, due to the lack of sufficient preclinical and clinical trial data across different cancer types. Second, the radiotherapy equipment currently capable of delivering FLASH-RT beams is not widely available, highlighting the need to upgrade existing systems or develop innovative new accelerators. Third, key treatment parameters—such as total dose, dose rate, and fractionation—have yet to be fully established. Fourth, the underlying mechanism of the FLASH effect remains poorly understood, representing a major barrier to its advancement. Before FLASH-RT can become a viable clinical option, a deeper understanding of the physicochemical and biological basis of the FLASH effect is essential.

There are several hypotheses proposed to explain the FLASH effect. The main hypothesis is the oxygen depletion mechanism, where oxygen levels surrounding the irradiated cells are rapidly depleted resulting in the formation of a hypoxic environment enhancing the radiation resistance of the cells (Yan *et al.*, 2024 ▸). Other proposed mechanisms include the radical interaction hypothesis where FLASH-RT results in the formation of free radicals such as reactive oxygen species (ROS), which in turn causes damage to the cell’s DNA and lipids resulting in its death (Del Debbio *et al.*, 2023 ▸; Yan *et al.*, 2024 ▸). It has been proposed that FLASH-RT directly causes DNA damage as well as reducing the ability of the cell to repair such, resulting in the death of the cell (Adrian *et al.*, 2021 ▸; Del Debbio *et al.*, 2023 ▸; Yan *et al.*, 2024 ▸). It has been shown that FLASH-RT affects the body’s immune system as well as causes remodelling to the tumour microenvironment, which may also contribute to its observed *in vivo* effects (Yan *et al.*, 2024 ▸).

The majority of research involving FLASH-RT is based on using charged particle beams (electrons and protons), while only a few have been based on X-rays (Adrian *et al.*, 2022 ▸; Guo *et al.*, 2025 ▸). The reason for this discrepancy is due to the absence of X-ray sources such as synchrotrons which can deliver UHDR beams.

It has been shown in a number of studies that the addition of gold nanoparticles enhances the cytotoxic effect of radiation on cells (Chithrani *et al.*, 2010 ▸; Engelbrecht-Roberts *et al.*, 2025 ▸; Guo *et al.*, 2025 ▸; Rahman *et al.*, 2009 ▸; Shahhoseini *et al.*, 2019 ▸; Shahhoseini *et al.*, 2021 ▸). Part of this is due to the Auger effect where electrons released from irradiated metal atoms such as gold (Au) can cause DNA and membrane damage both of which can be lethal to the cell (Ku *et al.*, 2019 ▸). In melanoma cells we observed a greater increase in the killing of these cells exposed to UHDR compared with those exposed to low dose rate radiation. We also examined whether hypoxia played a significant role in an X-ray generated FLASH effect, by exposing human epithelial melanocytes and colon cells along with their cancer derivatives to relatively low dose rate and UHDR beams. Using the different X-rays generated at the synchrotron, we assessed variations in cell viability under normoxic and hypoxic conditions in these matched cell lines to enhance our understanding of the effect FLASH-RT has on cancerous and non-cancerous cells and to what extent this is influenced by the oxygenated status of the cells. We observed that following exposure to >10 Gy the UHDR beams generated a greater cytotoxic effect on the tumour cells compared with their non-cancerous counterparts, while hypoxia was shown to confer a protective effect.

## 2. Materials and methods

### 2.1. Cell culture and cell lines

Human colon epithelial cells CCD841 (ATCC CRL-1790) and epithelial melanocytes (HEM) (ATCC PCS-200-012) cells were purchased from Invitro Technologies (Melbourne, Australia). CCD841 cells were grown in Eagle’s minimum essential medium (EMEM, Sigma-Aldrich, St Louis, USA) supplemented with 10% FBS (NZ Gibco FBS, Thermo Fisher Scientific, Waltham, USA). HEM were grown in melanocyte growth medium plus growth supplement (Cell Applications Inc., San Diego, USA). CaCo2 (colorectal adenocarcinoma) and MM96L (secondary melanoma isolated from lung tissue) cells were grown in RPMI medium (Gibco, Thermo Fisher Scientific) with 10% FBS under aseptic conditions at 37°C in a humidified atmosphere containing 5% CO_2_. Cells were harvested from 75 cm^2^ tissue culture flasks using TrypLE Express (Thermo Fisher Scientific) and resuspended in culture medium at 10^6^ cells ml^−1^. Aliquots (0.1 ml) of the cell suspension were added to sterile 0.2 ml PCR tubes. Three tubes were irradiated for each dose and condition tested.

### 2.2. Hypoxic conditions

For hypoxic conditions cells in 0.2 ml PCR tubes were sealed in a plastic bag with the lids open with an oxygen scavenger (AnaeroPack, Mitsubishi Gas Chemical, Tokyo, Japan) and oxygen meter (Bakmiwewa *et al.*, 2015 ▸). The tubes were incubated at 37^o^C until the oxygen level was approximately 1% (15 to 30 min). The tubes were closed *in situ* before the bag was opened and the cells irradiated and then reoxygenated within 30 min.

### 2.3. Gold nanoparticles

MM96L melanoma cells were used to investigate whether gold nanoparticles (AuNPs) enhanced the cytotoxic effect of UHDR beams. Cells grown in 25 cm^2^ flasks were treated with 1 m*M* AuNPs (AuroVist^TM^ 15 nm, Nanoprobes, Yaphank, USA) for 24 h. The cells were then harvested from the flasks, using TrypLE Express, and resuspended in culture medium at 10^6^ cells ml^−1^. Aliquots (0.1 ml) of the cell suspension were added to sterile 0.2 ml PCR tubes. Three tubes were irradiated for each dose. Cell viability was determined 72 h post-irradiation using the MTS assay.

### 2.4. MTS assay

After irradiation, the cells were diluted in their respective tissue culture medium containing 50 U ml^−1^ penicillin and 50 mg ml^−1^ streptomycin (Gibco, Thermo Fisher Scientific), and 5 × 10^3^ cells in 0.2 ml were added per well in a 96 well plate. The cells were incubated at 37^o^C in 5% CO_2_ for 24, 48 or 72 h. At the end of this period, 20 µL of a 10% (*v*/*v*) MTS solution [3-(4,5-di­methyl­thia­zol-2-yl)-5-(3-carb­oxy­meth­oxy­phenyl)-2-(4-sulfo­phenyl)-2H-tetrazolium] (MTS assay kit, Abcam, Cambridge, UK) was added to the wells. Absorbance readings at 490 nm were recorded after 1 to 4 h incubation at 37°C (average of five wells).

### 2.5. Reference dosimetry

The experiments were performed in Hutch 2B on the Imaging and Medical Beamline (IMBL) at the ANSTO Australian Synchrotron (Clayton, Australia) (Livingstone *et al.*, 2017 ▸). The cells were exposed to different doses of X-rays delivered in the IMBL. The IMBL allows a selection of differing dose rates and spectra via alteration of the magnetic field of the superconducting multi-pole wiggler (SCMPW) and in-vacuum filtration. For these experiments, the beamline configuration was 4T-CuCu (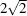 mm Cu dominant in-vacuum filtration) and 1.4T-CuCu to achieve dose rates of ∼500 Gy s^−1^ and ∼3 Gy s^−1^, respectively. These dose rates were chosen to represent two distinct conditions of FLASH and non-FLASH dose deliveries. It should be noted that ∼3 Gy s^−1^ is the lowest achievable dose rate for this beam quality in the IMBL, which is higher than the dose rate used in clinical CONV-RT.

**Figure 1:**
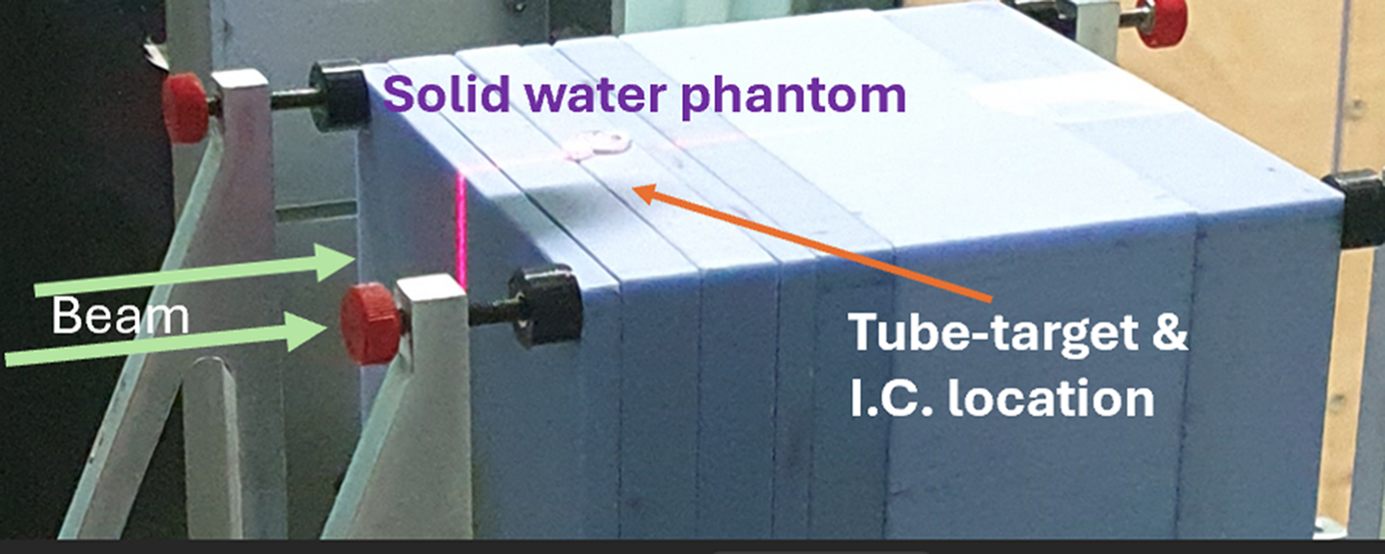
Experimental setup for dose delivery to a sample. The green arrows show the direction of the incident beam. The phantom (100 mm × 100 mm × 100 mm) is made of solid water material. The tube containing the cells was placed in the block at a depth of a 20 mm, relative to the incident beam. On the downstream face of the solid water phantom (not shown in the picture), a sheet of EBT4 GafChromic film was placed, to qualitatively verify dose delivery post-irradiation.

Reference dosimetry was performed for both beamline configurations using a 2.019 mm × 20 mm intrinsic beam and a treatment field of 20 mm × 20 mm incident on the small animal dynamic MRT stage. Measurements were taken using a PTW PinPoint (31022) ionization chamber (PTW, Freiburg, Germany) at 20 mm depth in a 100 mm × 100 mm × 100 mm solid water HE phantom (Model 557, Gammex Inc, Wisconsin, USA) (Fig. 1 ▸).

The dose rate generated by the beam passing through the 4 T magnetic field then Cu/Cu filtration can reach >500 Gy s^−1^ while for those generated at 1.4 T it can be as low as 2.8 Gy s^−1^. These dose rates represent two distinct conditions of FLASH and non-FLASH dose deliveries.

Dose was delivered to the target using the dynamic MRT stage according to equation (1),
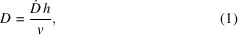
where *D* is the delivered dose (Gy), 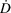 is the dose rate (Gy s^−1^), *h* the height of the intrinsic beam (mm) and *v* the velocity of stage translation (mm s^−1^). Correction factors were applied to compensate recombination, polarity, temperature and pressure, beam quality, and electrometer calibration according to Fournier *et al.* (2016 ▸). The dose rates measured under reference conditions were 250 Gy s^−1^ and 2 Gy s^−1^ for the 4T-CuCu and 1.4T-CuCu conditions, respectively. The limiting factor for minimum deliverable dose in each configuration was the maximum speed of the stage (20 mm s^−1^) and the height of the intrinsic beam. The chosen doses to be delivered to the samples (in both beamline configurations) were 10 Gy and 25 Gy, both of which were achievable within the limits of the dynamic MRT stage.

### 2.6. Dose delivery

Samples were positioned in the same bore that accepts the ionization chamber to match reference dosimetry conditions. The bore is filled with water to eliminate air gaps when the sample tubes are inserted. This allows for a simple and replicable setup between experiments.

During irradiation, the exit dose on the downstream face of the phantom was monitored using EBT4-type GafChromic film (Ashland Inc. Wilmington, USA) as qualitative quality assurance that dose delivery was successful.

### 2.7. Data and statistical analysis

Data are expressed as the mean ± standard error of the mean (SEM) of five replicate samples. Statistical comparisons were made using unpaired Student *t*-tests (GraphPad Prism Version 10.0 for Macintosh, GraphPad Software, San Diego, USA). Statistical significance was defined as *p* < 0.05.

## 3. Results and discussion

These results comprise the effects of low and high dose rate X-ray beams on normal and their cancer derivative cells. Two cell types are investigated in this study: epithelial melanocytes and colon cells as well as their respective cancerous counterparts.

HEM and their cancerous derived melanoma cells (MM96L) were investigated in this study. MM96L are secondary melanoma cells isolated from lung tissue and possess the BRAF^V600E^ mutation, while HEM lack this mutation and are defined as being BRAF wild type (BRAF^WT^) cells (Muthusamy & Piva, 2013 ▸). The other cell types investigated in this study were human colon epithelial cells (CCD841) and their cancerous derivative CaCo2 cells (Rombouts *et al.*, 2021 ▸). We chose these two cell types as they represent cells that are located externally (HEM and MM96L) or internally (CCD841 and CaCo2) in the body. The rationale for this choice was to observe whether the FLASH effect is similar irrespective of the anatomical origin of the cells.

**Figure 2:**
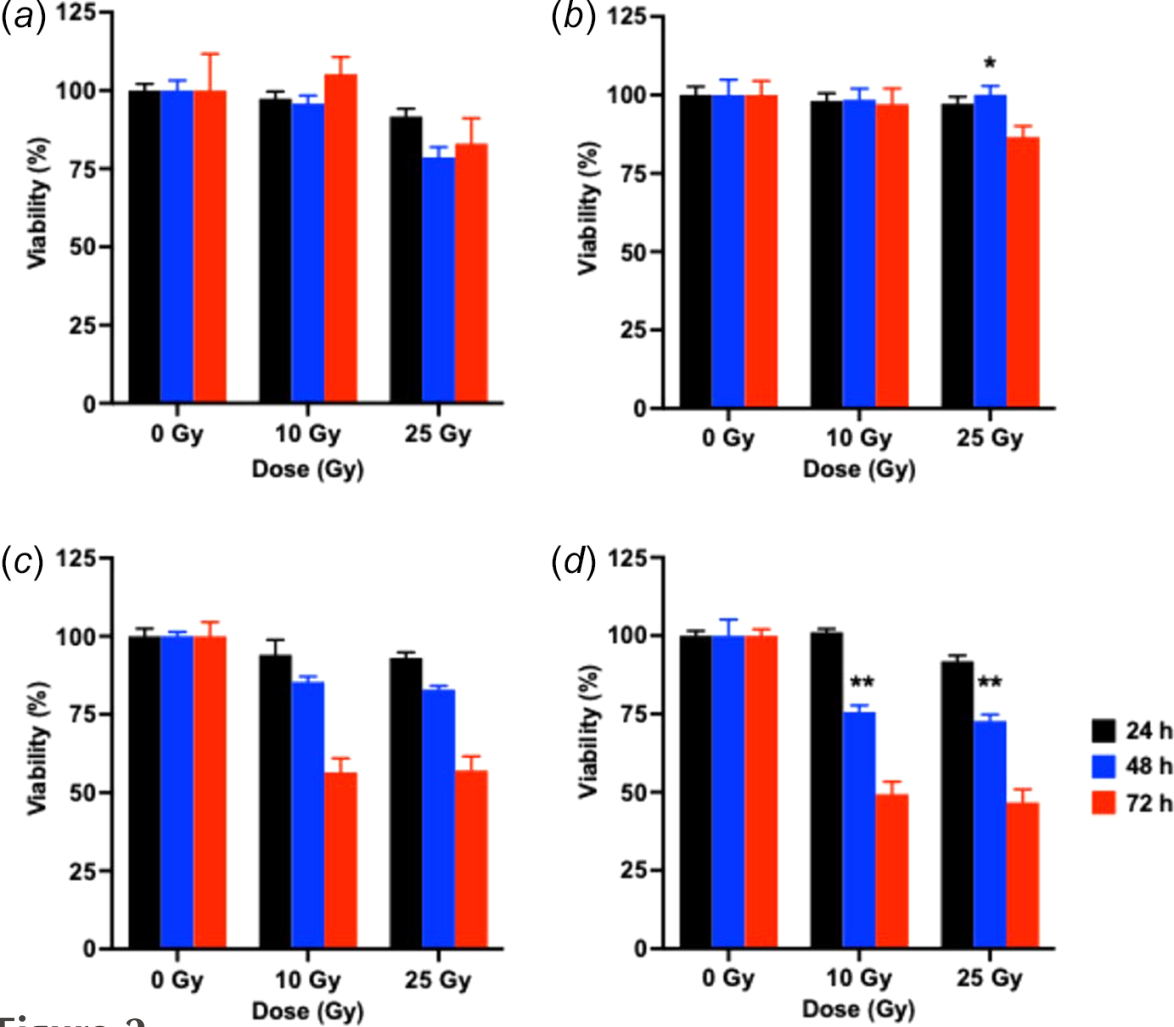
Comparing the effect of low dose rate and UHDR on the viability of HEM and MM96L cells. Cells were exposed to either 0, 10 or 25 Gy of radiation and cell viability determined after 24, 48 and 72 h. HEM cells were exposed to low dose rate radiation (*a*) and UHDR (*b*), while MM96L cells were exposed to low dose radiation (*c*) and UHDR (*d*). Cell viability is expressed as a percentage of that of the unirradiated control cells (represented as 100%) at that time point. Results expressed are the mean ± SEM (*n* = 5). Differences between those cells exposed to low dose rate or UHDR radiation (10 or 25 Gy) are shown as *p < 0.01 and **p < 0.001.

HEM cells were found to be more sensitive to higher doses (10 and 25 Gy) of low dose rates of X-ray radiation at 48 and 72 h post-irradiation than when these cells were exposed to UHDRs [Figs. 2 ▸(*a*) and 2(*b*)]. Exposure to 10 Gy radiation at either dose rate had no significant dose rate effect on melanocyte cell viability over 72 h, with a slight increase in cell number observed at 72 h in cells exposed to low dose X-rays. However, when the cells were exposed to 25 Gy radiation, the viability of cells exposed to low dose rate radiation X-rays had a lower viability at 48 and 72 h compared with those cells exposed to UDHR rates.

A different effect was observed for the melanoma cells. MM96L cells were shown to be more sensitive to both low dose and UHDR radiation over this 72 h period compared with the non-cancerous melanocytes [Figs. 2 ▸(*c*) and 2(*d*)]. When these cells were exposed to UHDR compared with low dose rate X-rays there was a significant loss (*p* < 0.001) of viability of the MM96L cells exposed to 10 and 25 Gy at 48 h [Fig. 2 ▸(*d*)]. We observed that increasing the exposure to higher levels of radiation had no impact on the survival of these cells between 48 and 72 h following exposure to either low dose rate X-rays (57% versus 57% for 10 and 25 Gy, respectively) or UHDRs (49% versus 47% for 10 and 25 Gy, respectively). Exposure to UHDR X-rays caused ∼10% loss in the viability of MM96L cells compared with low dose rate X-rays when the cells were exposed to the same radiation dose (10 or 25 Gy).

These effects are clearly showing that the high dose rate effects were less pronounced in HEM compared with MM96L cells when these cells were exposed to UHDR X-ray beams. Of interest was that low dose X-rays caused a greater loss of cell viability at 25 Gy in HEM than when these cells were exposed to UDHR. This effect was not observed in the MM96L cells where both low dose and UDHR X-rays triggered cell death at both doses and time points. This shows the dose rate effects to be consistent with the FLASH effect which has been validated in tissue and animal studies (Adrian *et al.*, 2021 ▸; Adrian *et al.*, 2022 ▸; Del Debbio *et al.*, 2025 ▸; Yan *et al.*, 2024 ▸). It has been documented lately that the FLASH effect varies in its impact between different cell types as measured using spheroids (Dela *et al.*, 2026 ▸).

**Figure 3:**
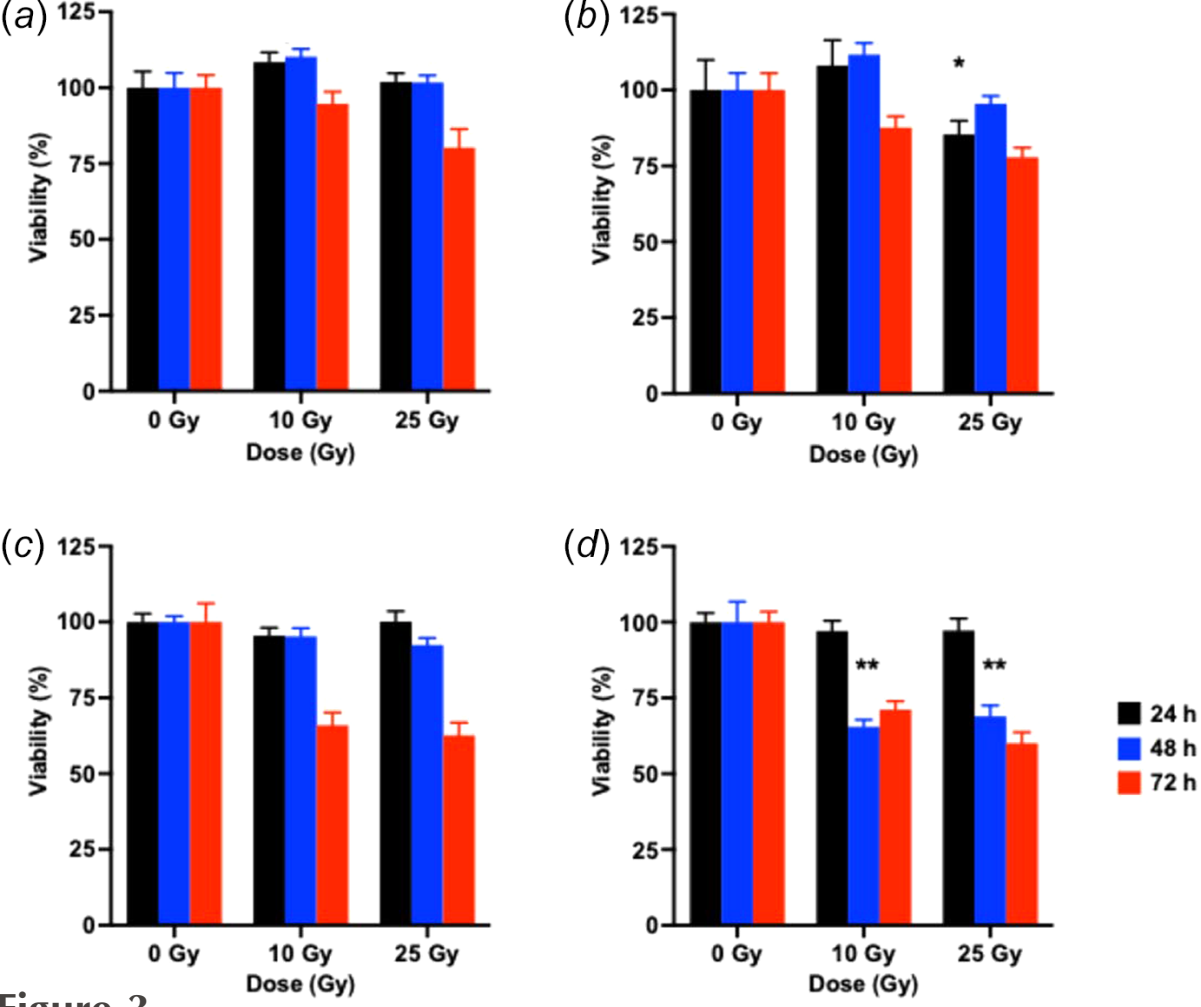
Comparing the effect of low dose rate and UHDR on the viability of CCD841 and CaCo2 cells. Cells were exposed to either 0, 10 or 25 Gy of radiation and cell viability determined after 24, 48 and 72 h. CCD841 cells were exposed to low dose radiation (*a*) and UHDR (*b*), while CaCo2 cells were exposed to low dose radiation (*c*) and UHDR (*d*). Cell viability is expressed as a percentage of that of the unirradiated control cells (represented as 100%) at that time point. Results expressed are the mean ± SEM (*n* = 5). Differences between those cells exposed to low dose rate or UHDR radiation (10 or 25 Gy) are shown as *p < 0.05 and **p < 0.001.

When the CCD841 (colon epithelial cells) were exposed to low dose rate and UHDR X-rays a similar result to that seen for HEM cells was observed [Figs. 3 ▸(*a*) and 3(*b*)]. At high dose 10 and 25 Gy similar responses were observed over 72 h in these cells exposed to both forms of X-ray dose rates except for cells exposed to 25 Gy UHDR at 24 h where cell survival was significantly lower (*p* < 0.05) [Fig. 3 ▸(*b*)]. This result shows that in non-cancerous cells there is no observed differences between FLASH and lower-than-FLASH dose rate beams which is similar to that seen in other studies where such/similar cells have been exposed to both CONV-RT and UHDR-RT (Adrian *et al.*, 2021 ▸; Del Debbio *et al.*, 2025 ▸).

The results observed when CaCo2 (colon carcinoma) cells were exposed to both dose rate types of X-rays were different from that seen for CCD841 cells [Figs. 3 ▸(*c*) and 3(*d*)]. CaCo2 cells were shown to be more sensitive to both low dose rate and UHDR radiation over 72 h compared with the non-cancerous CCD841 cells. When the CaCo2 cells were exposed to 10 and 25 Gy of UHDR X-rays there was significant loss (*p* < 0.001) of cell viability (∼30%) at 48 h when compared with the same cells exposed to the same doses of low dose rate X-rays. After 72 h the difference was minimal when the effects of low dose rate (66% and 63% for 10 and 25 Gy, respectively) and UDHR X-rays (71% and 60% for 10 and 25 Gy, respectively) on these cells was compared. Exposure to UDHR at 48 h and at lower doses is shown to be more effective in killing CaCo2 cells than from radiation delivered of the same dose at much lower dose rate, which is what has been observed elsewhere as part of the FLASH effect on tumour cells (Adrian *et al.*, 2021 ▸; Böhlen *et al.*, 2023 ▸; Del Debbio *et al.*, 2025 ▸; Yan *et al.*, 2024 ▸).

The effect of hypoxia on the viability of the irradiated melanocytic (HEM and MM96L) cells was examined by depleting the level of oxygen in these cells before exposing them to both beam types (low dose rates and UHDR). The sterile microfuge tubes containing the cells were placed inside a plastic bag with the lids open along with an oxygen scavenger and meter to detect the % oxygen present. When the O_2_ levels fell to ∼1% the tubes were sealed (as described in the *Methods*[Sec sec2] section), and these hypoxic cells were irradiated in the same way as the normoxic cells.

**Figure 4:**
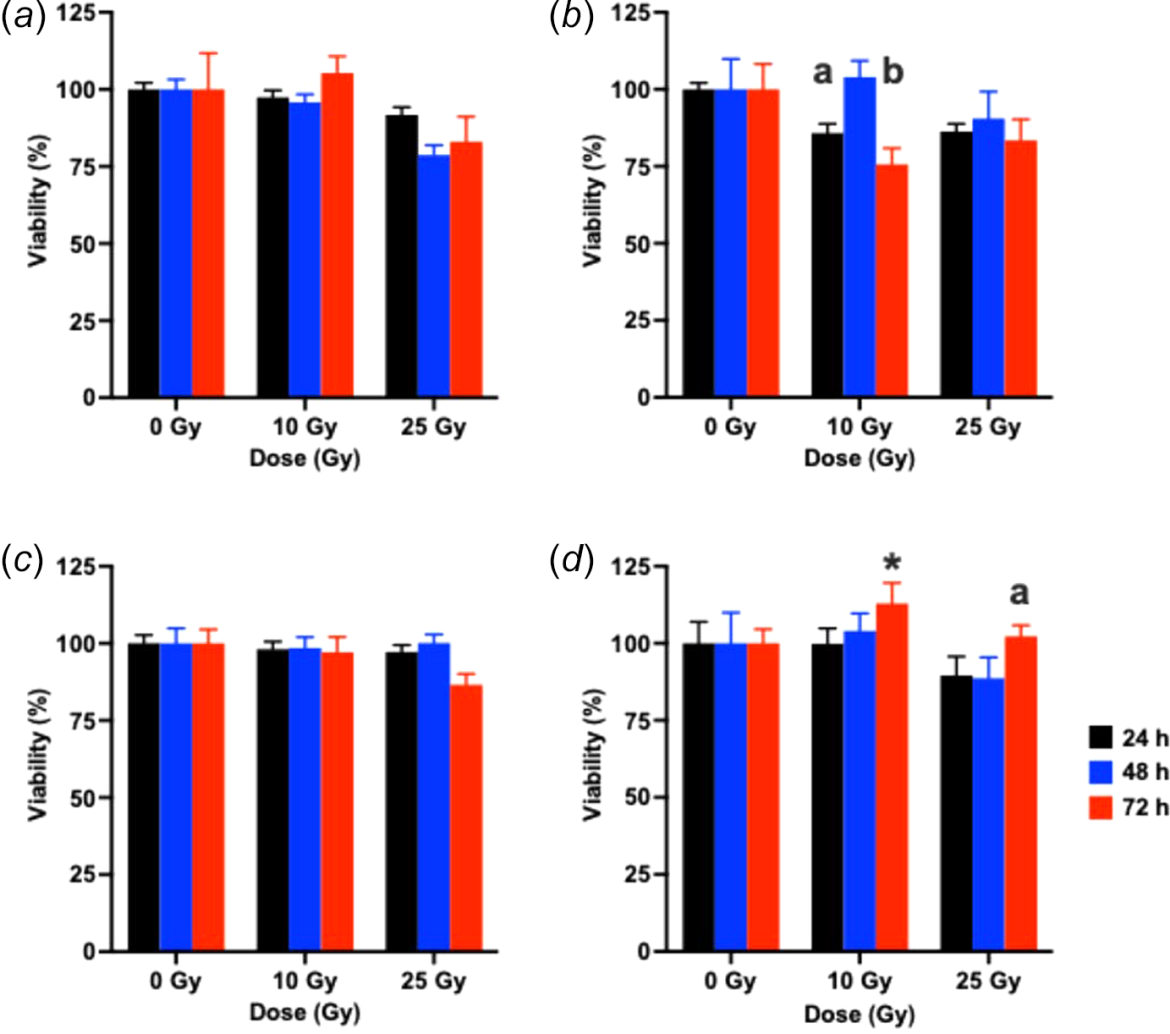
Comparing the effect of low dose rate and UHDR on the viability of hypoxic HEM cells. Cells were exposed to either 0, 10 or 25 Gy of radiation and cell viability determined after 24, 48 and 72 h. Normoxic HEM (*a*) and hypoxic HEM (*b*) were exposed to low dose radiation, while normoxic HEM (*c*) and hypoxic HEM (*d*) were exposed to UHDR. Cell viability is expressed as a percentage of that of the unirradiated control cells (represented as 100%) at that time point. Results expressed are the mean ± SEM (*n* = 5). Differences between those cells exposed to low dose rate or UHDR radiation (10 or 25 Gy) are shown as *p < 0.01, while differences due to the oxygenated state of the cell exposed to the same dose and dose rate are shown as ^a^*p* < 0.05 and ^b^*p* < 0.01.

The normoxic HEM cells were shown to be significantly less sensitive to high doses (10 Gy) of low dose rate radiation after 24 and 72 h post-irradiation (*p* < 0.05 and *p* < 0.01, respectively) than were the corresponding hypoxic cells except at 72 h following exposure to 25 Gy (*p* < 0.01) [Figs. 4 ▸(*a*) and 4(*b*)]. When these cells were exposed to high doses (10 and 25 Gy) of UHDR, cell survival rates were similar between the normoxic and hypoxic cells at both time points [Figs. 4 ▸(*c*) and 4(*d*)]. We observed that there was a higher survival of both normoxic or hypoxic HEM cells exposed to UHDR when compared with cells irradiated at low dose rates. The most significant enhancement of cell survival was seen at 72 h for those cells exposed to 25 Gy (*p* < 0.05).

**Figure 5:**
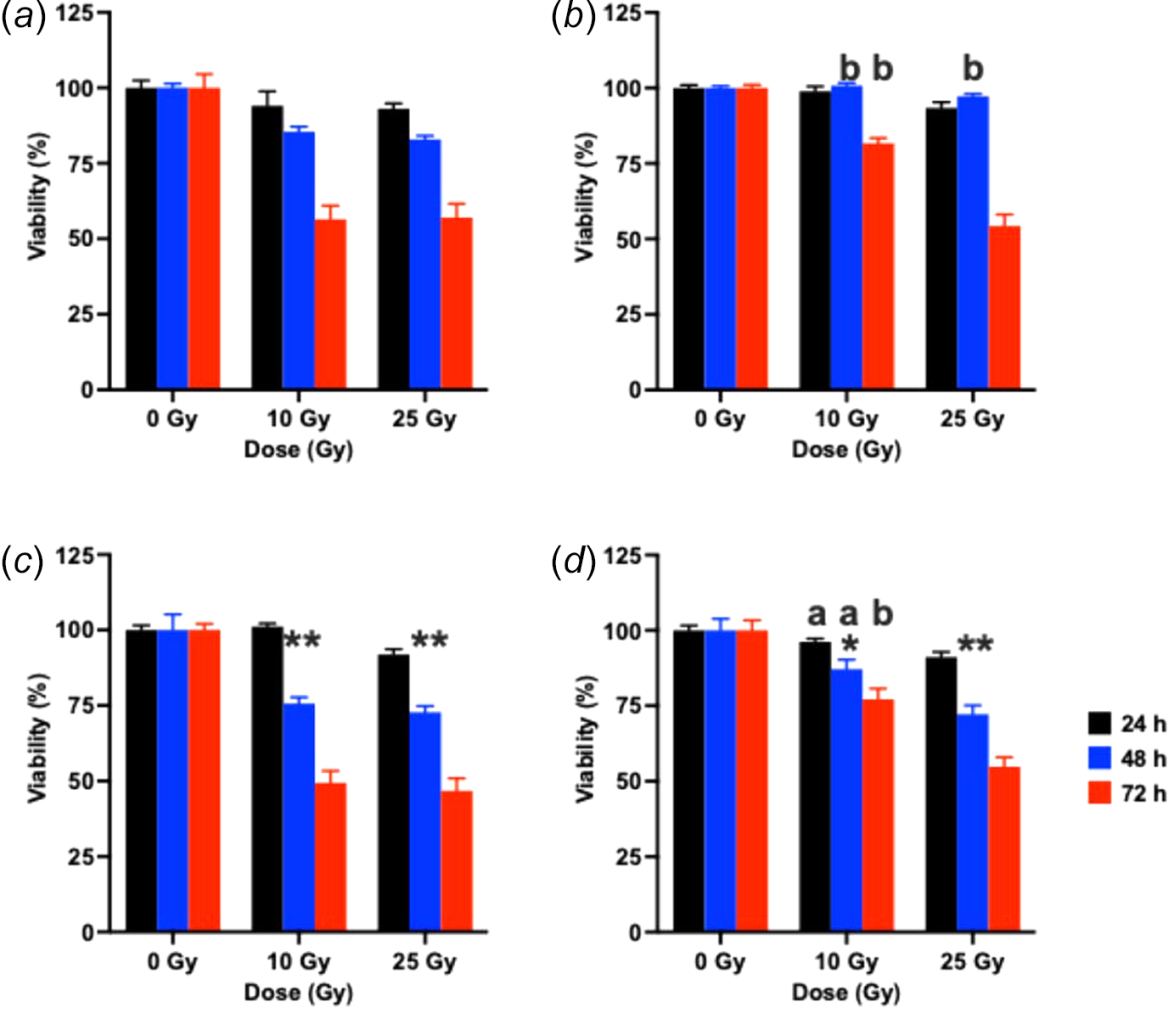
Comparing the effect of low dose rate and UHDR on the viability of hypoxic MM96L cells. Cells were exposed to either 0, 10 or 25 Gy of radiation and cell viability determined after 24, 48 and 72 h. Normoxic (*a*) and hypoxic MM96L cells (*b*) were exposed to low dose radiation, while normoxic (*c*) and hypoxic MM96L cells (*d*) were exposed to UHDR. Cell viability is expressed as a percentage of that of the unirradiated control cells (represented as 100%) at that time point. Results expressed are the mean ± SEM (*n* = 5). Differences between those cells exposed to low dose rate or UHDR radiation (10 or 25 Gy) are shown as *p < 0.01 and **p < 0.001, while differences due to the oxygenated state of the cell exposed to the same dose and dose rate are shown as ^a^*p* < 0.05 and ^b^*p* < 0.001.

MM96L cells exposed to high doses (10 and 25 Gy) of low dose rate X-rays caused significant (*p* < 0.001) cell death in normoxic MM96L cells at 72 h but not at 48 h [Fig. 5 ▸(*a*)]. However, the same effect was not observed in the hypoxic cells at either time point [Fig. 5 ▸(*b*)]. Cell survival was significantly higher (*p* < 0.001) at 48 h in the hypoxic cells exposed to 10 or 25 Gy low dose radiation than in the normoxic cells. At 72 h the survival of MM96L cells exposed to 25 Gy radiation (55% versus 53% for hypoxic and normoxic cells, respectively) was similar; those exposed to 10 Gy were much higher (∼25%) in the hypoxic cells compared with normoxic cells.

As seen earlier when normoxic MM96L cells are exposed to UHDR at 10 or 25 Gy, cell viability significantly (*p* < 0.001) fell at 48 h [Fig. 5 ▸(*c*)]; however, the viability of these cells was less than those cells treated with low dose rate radiation. When the hypoxic MM96L cells were exposed to 10 Gy UHDR [Fig. 5 ▸(*d*)] there was a significant reduction (*p* < 0.05) in the viability of these cells compared with those cells exposed to low dose rate radiation (72% versus 97% viability, respectively) [Figs. 5 ▸(*c*) and 5(*d*)]. However, when these cells were exposed to 25 Gy radiation, similar levels of viability were seen irrespective of the dose rate. When the MM96L cells were exposed to 10 Gy of UDHR the hypoxic cells were less sensitive at all times (24, 48 and 72 h, *p* < 0.05, *p* < 0.05 and *p* < 0.001, respectively) compared with the normoxic cells; however, at the higher dose (25 Gy) no differences were observed. This result was similar to that observed when DU145 prostate cancer cells were exposed to UDHR under normoxic and hypoxic conditions and clearly shows that a FLASH effect has occurred in these cells (Adrian *et al.*, 2020 ▸).

It has been suggested by many researchers that the level of intracellular oxygen is crucial for the observed FLASH effect (Adrian *et al.*, 2020 ▸; Adrian *et al.*, 2022 ▸; Del Debbio *et al.*, 2023 ▸; Yan *et al.*, 2024 ▸). Other reasons for the observed FLASH effect have also been suggested including direct damage to the cell’s DNA, DNA damage caused by ROS formation, as well as the remodelling of the tumour microenvironment *in vivo* (Del Debbio *et al.*, 2023 ▸; Yan *et al.*, 2024 ▸). In this study we exposed adhered cells in suspension to both CONV-RT and UHDR-RT, in a normoxic or hypoxic state. Following radiation, the cells were allowed to adhere and after 24, 48 or 72 h in culture the cell viability was assessed. While other studies have used adhered cells grown in culture flasks (Adrian *et al.*, 2021 ▸; Adrian *et al.*, 2020 ▸; Del Debbio *et al.*, 2025 ▸), we also observed the FLASH effect using adhered cells in suspension exposed to X-ray radiation generated by a beamline at a synchrotron.

**Figure 6:**
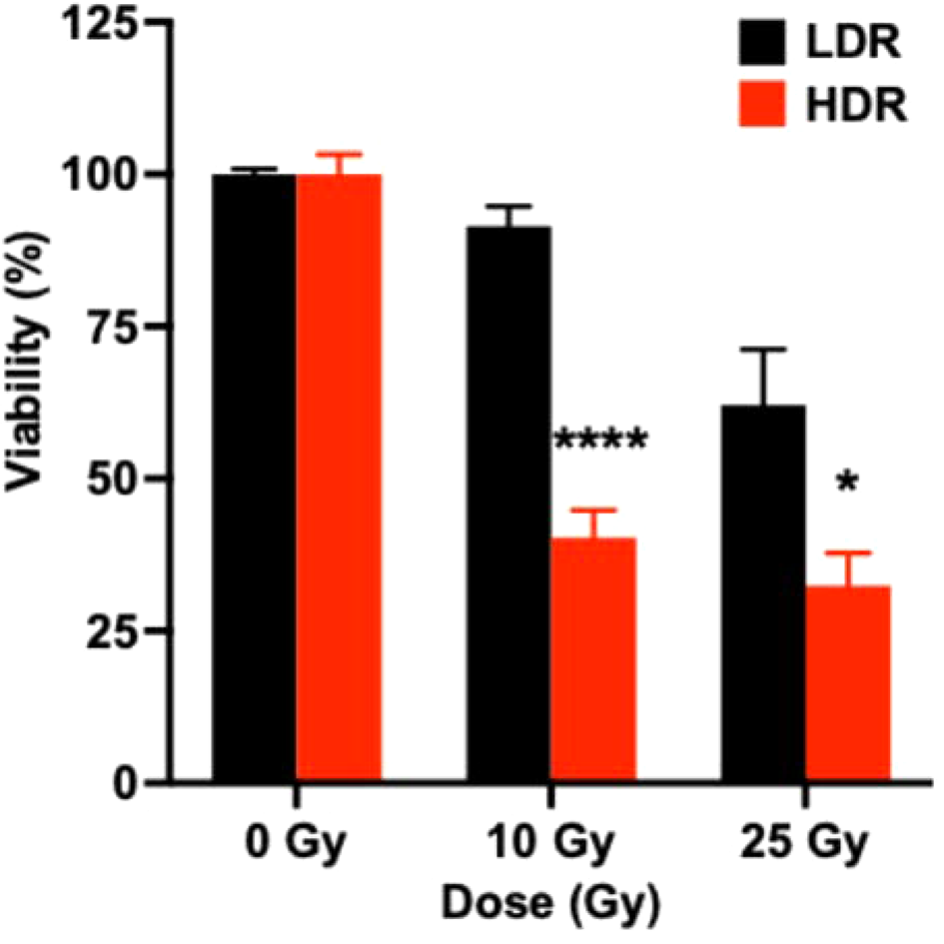
Comparing the effect of low dose rate and UHDR on the viability of MM96L cells treated with AuNPs. Cells were pretreated with 1 m*M* AuNPs for 24 h before being exposed to either 0, 10 or 25 Gy of radiation and cell viability determined after 72 h. Cell viability is expressed as a percentage of that of the unirradiated control cells (represented as 100%) at that time point. Results expressed are the mean ± SEM (*n* = 5). Differences between those cells exposed to low dose rate or UHDR radiation (10 or 25 Gy) are shown as *p < 0.05 and ****p < 0.0001.

We have shown previously that AuNPs have been shown to enhance the cytotoxic effect of radiation on cultured cells (Rahman *et al.*, 2009 ▸; Shahhoseini *et al.*, 2019 ▸; Shahhoseini *et al.*, 2021 ▸). We pretreated MM96L cells with 1 m*M* AuNPs for 24 h before exposing the cells to either 10 or 25 Gy low dose rate or UHDR X-rays and cell viability was measured 72 h later (Fig. 6 ▸). Cells exposed to UHDR at both 10 (*p* < 0.0001) and 25 Gy (*p* < 0.05) were less viable than those cells exposed to low dose rate radiation. This suggests that AuNPs did enhance cell killing at the higher dose rate.

The effect of the AuNPs resulted in a ∼10% reduction in the viability of those cells exposed to UHDR irrespective of the total dose administered (40.34 ± 4.49% versus 49.42 ± 3.92% at 10 Gy and 32.44 ± 5.36% versus 46.75 ± 4.16% at 25 Gy, respectively). The enhanced rate of cell killing observed could be due to the Auger effect generated by these gold nanoparticles (Antosh *et al.*, 2015 ▸; Engelbrecht-Roberts *et al.*, 2025 ▸); however, further experiments are needed to confirm this. We wish to undertake further experiments to observe the effect these treatments have on cell signalling, intracellular ROS generation and cell death pathways to enhance our understanding of the FLASH effect and its effect on tumour cells.

## 4. Conclusions

In this research four cell lines (two human primary cell lines, one found on the skin and the other internally as well as a respective cancerous derivative) were exposed to radiation doses (mainly 10 and 25 Gy) either delivered as UHDR or low dose rate X-rays from the IMBL at the Australian Synchrotron. The adhered cells were suspended prior to being exposed to X-ray radiation, after which they were allowed to reattach into a well in a culture plate and viability measured 24, 48 or 72 h post-irradiation. Some cells were depleted of oxygen prior to irradiation to observe if oxygen plays a major role in the FLASH effect. We observed almost no differences between the non-cancerous cells exposed to 10 or 25 Gy radiation delivered either via low dose rates or UHDRs as predicted by the FLASH phenomenon. However, both cancerous cell lines were ∼30% more sensitive to the effect of UHDR than compared with low dose rates. We have shown that adhered cells in suspension also exhibit a FLASH effect similar to that seen in previous studies using different adhered cells that have been exposed to electron and positron beams (Adrian *et al.*, 2021 ▸; Adrian *et al.*, 2022 ▸; Del Debbio *et al.*, 2025 ▸; Guo *et al.*, 2025 ▸; Yan *et al.*, 2024 ▸).

We also observed the effect anoxia had on the melanocyte-derived cells. The results obtained from this study were similar to an earlier study (Adrian *et al.*, 2020 ▸) and support the role oxygen may play in the FLASH effect. Preliminary experiments using AuNPs were shown to enhance the FLASH effect as well. We intend to further examine the effect AuNPs as well as that of chemotherapeutic agents to cells exposed to UHDR to observe whether these combinations of treatments selectively enhances the killing of tumour cells.

## Data Availability

Data supporting the results shown in the manuscript are available upon request from the corresponding author.
